# The domestic cat as a natural animal model of Alzheimer’s disease

**DOI:** 10.1186/s40478-015-0258-3

**Published:** 2015-12-10

**Authors:** James K. Chambers, Takahiko Tokuda, Kazuyuki Uchida, Ryotaro Ishii, Harutsugu Tatebe, Erika Takahashi, Takami Tomiyama, Yumi Une, Hiroyuki Nakayama

**Affiliations:** Department of Veterinary Pathology, Graduate School of Agricultural and Life Sciences, The University of Tokyo, Tokyo, Japan; Department of Molecular Pathobiology of Brain Diseases, Kyoto Prefectural University of Medicine, Kyoto, Japan; Department of Neurology, Kyoto Prefectural University of Medicine, Kyoto, Japan; Laboratory of Veterinary Pathology, School of Veterinary Medicine, Azabu University, Kanagawa, Japan; Department of Neuroscience, Osaka City University Graduate School of Medicine, Osaka, Japan

**Keywords:** Alzheimer’s disease (AD), Amyloid β (Aβ), Cat, Neurodegeneration, Oligomer, Tau

## Abstract

**Introduction:**

Alzheimer’s disease (AD) is the most dominant neurodegenerative disorder that causes dementia, and no effective treatments are available. To study its pathogenesis and develop therapeutics, animal models representing its pathologies are needed. Although many animal species develop senile plaques (SP) composed of amyloid-β (Aβ) proteins that are identical to those found in humans, none of them exhibit neurofibrillary tangles (NFT) and subsequent neurodegeneration, which are integral parts of the pathology of AD.

**Results:**

The present study shows that Aβ accumulation, NFT formation, and significant neuronal loss all emerge naturally in the hippocampi of aged domestic cats. The NFT that form in the cat brain are identical to those seen in human AD in terms of their spatial distribution, the cells they affect, and the tau isoforms that comprise them. Interestingly, aged cats do not develop mature argyrophilic SP, but instead accumulate intraneuronal Aβ oligomers in their hippocampal pyramidal cells, which might be due to the amino acid sequence of felid Aβ.

**Conclusions:**

These results suggest that Aβ oligomers are more important than SP for NFT formation and the subsequent neurodegeneration. The domestic cat is a unique animal species that naturally replicates various AD pathologies, especially Aβ oligomer accumulation, NFT formation, and neuronal loss.

**Electronic supplementary material:**

The online version of this article (doi:10.1186/s40478-015-0258-3) contains supplementary material, which is available to authorized users.

## Introduction

Alzheimer’s disease (AD) is a neurodegenerative disorder characterized by three major pathologies: senile plaques (SPs), neurofibrillary tangles (NFTs), and neuronal loss. The former two are extracellular and intracellular argyrophilic aggregates composed of amyloid β (Aβ) and hyperphosphorylated tau protein, respectively. Accumulating evidence indicates that Aβ accumulation leads to NFT formation and subsequent neuronal loss and cognitive dysfunction [24, 29, 47]. Based on this notion, various transgenic (Tg) mouse models have been generated by introducing human *APP* (amyloid precursor protein) or *PSEN* (presenilin) with the mutations linked to familial AD [23]. These Tg mice produce human Aβ beyond physiological levels, leading to massive formation of SPs [13]. Nevertheless, they fail to develop NFTs and neuronal loss unless mutant *MAPT* (tau) is simultaneously introduced. Although wild-type mice do not spontaneously form SPs or NFTs, many other animal species such as monkeys and dogs are known to develop SPs as they age [19, 25, 44, 48, 58]. However, these animals do not display NFTs and neuronal loss. If an animal species can be identified that naturally develops SPs, NFTs, and neuronal loss, it could be a desirable animal model for translational studies of AD. Hyperphosphorylated tau (AT8-positive) has been observed in the brains of domestic cats with signs of neurological dysfunction [18, 26]. Furthermore, recently, we found that aged leopard cats and cheetahs display both Aβ deposits and NFTs in the brains [8, 49]. Leopard cats and cheetahs are endangered wild animals, thus they are not adequate for further laboratory examination. In contrast, domestic cats, which diverged from a common ancestor with the leopard cat and cheetah approximately 6.2 million years ago [32], could be used in such studies if they produce Aβ deposits and NFT. Here, we report that aged domestic cats naturally accumulate Aβ oligomers, produce NFT, and moreover suffer hippocampal neuronal loss, and thus, could serve as a valuable animal model of human AD.

## Materials and methods

### Brain samples

Cat brain tissues of various ages were examined (Table 1). All the adult cat brains were obtained through routine necropsies performed at the Department of Veterinary Pathology, the University of Tokyo. The fetal brain samples were purchased from a laboratory animal supplier (Nisseiken Co. Ltd., Tokyo, Japan). All procedures were done according to the institutional regulations for animal research. One hemisphere of the brain was fixed in 10 % phosphate-buffered formalin, and the other hemisphere was coronally sectioned and then frozen at −80 °C until use.

**Table 1:** Age, sex and immunohistochemical results for Aβ42 and hyperphosphorylated tau in cats

No.	Age	Sex	Aβ42	HP-tau
1	fetus (50 days)	F	−	+^a^
2	fetus (50 days)	M	−	+^a^
3	2-week-old	F	−	−
4	2-week-old	F	−	−
5	3-year-old	M	−	−
6	3-year-old	M	−	−
7	4-year-old	F	−	−
8	4-year-old	F	−	−
9	5-year-old	F	−	−
10	8-year-old	F	+	−
11	14-year-old	F	−	−
12	14-year-old	M	+	++
13	15-year-old	F	+	+
14	15-year-old	M	+	−
15	15-year-old	ND	+	−
16	16-year-old	F	+	−
17	16-year-old	M	+	−
18	17-year-old	F	+	−
19	17-year-old	F	+	+
20	17-year-old	ND	+	+
21	18-year-old	F	+	+
22	19-year-old	F	+	++
23	20-year-old	F	+	+
24	20-year-old	ND	+	++
25	22-year-old	M	+	+

Aβ42: -, negative; +, small aggregates of Aβ42 were observed in the parietal and temporal cortices on FA-pretreated sections, and intracellular aggregates of Aβ 42 were observed in the cytoplasm of hippocampal pyramidal cells. HP-tau: -, negative; +, AT8-positive cells limited to the entorhinal cortex; ++, AT8-positive cells in the entorhinal cortex and throughout the hippocampus. F, female; M; male, ND, no data; ^a^, weak AT8 positivity on the surface of cerebral cortex

### Histology

Formalin fixed paraffin-embedded tissues were cut into 4-μm-thick serial sections. The deparaffinized sections were then stained with HE, periodic acid-methenamine silver, Congo red and the Gallyas-Braak method. Digital images were obtained using an Olympus BX 50 microscope (Olympus, Tokyo, Japan) equipped with a Nikon DS-Ri1 digital camera (Nikon, Tokyo, Japan).

### Immunohistochemistry

Consecutive sections were stained using the immunoenzyme technique. Sections were deparaffinized and rehydrated. Antigen retrieval was done by heating or with FA (for Aβ). The sections were immersed in 1 % hydrogen peroxide in methanol for 20 min in order to deactivate endogenous peroxidases, and then immersed in 5 % skim milk in Tris-buffered saline (TBS) in order to avoid nonspecific binding of the antibody. The following primary antibodies were used: mouse anti-hyperphosphorylated-tau Ser202/Thr205 (clone AT8, 1:100, Thermo Scientific, Rockford, IL), mouse anti-hyperphosphorylated-tau Ser212/Thr214 (clone AT100, 1:100, Thermo Scientific), mouse anti-3-repeat-tau RD3 (clone 8E6/C11, 1:100, Millipore, Temecula, CA), mouse anti-4-repeat-tau RD4 (clone 1E1/A6, 1:100, Millipore), rabbit anti-pan-tau (1:100, Sigma, St. Louis, MO), mouse anti-Aβ42 (clone 12 F4, 1:1000, Millipore), rabbit anti-Aβ42 (1:1000, IBL, Gunma, Japan), rabbit anti-AβN1 (1:100, IBL), rabbit anti-AβpN3 (1:100, IBL), rabbit anti-Ubiquitin (1:200, Dako, Carpinteria, CA), rabbit anti-Apolipoprotein (Apo) E (A299, 1:100, IBL), and mouse anti-NeuN (clone A60, 1:100, Millipore). After incubation with each primary antibody at 4 °C overnight, immunolabeled antigens were visualized using the Dako Envision + System (Dako). In brief, the sections were incubated with the secondary antibody linked to a peroxidase-conjugated polymer backbone at 37 °C for 40 min, reacted with 0.05 % 3′3-diaminobenzidine plus 0.03 % hydrogen peroxide in Tris-hydrochloric acid buffer, and then counterstained with hematoxylin. Negative controls were obtained by omitting the primary antibodies. Neuronal loss in the pyramidal cell layer of the hippocampal CA1 region was evaluated by counting NeuN-positive cells displayed in Fig. 6a. Comparisons of the means among the three groups were performed with one-way ANOVA followed by Tukey's HSD test using SPSS software (IBM, Tokyo, Japan). Differences with a *P* value of <0.05 were considered significant.

### Double-labeling immunofluorescence

Sections were deparaffinized and rehydrated. Antigen retrieval was done by heating. In order to reduce autofluorescence, Sudan black B treatment was performed. Sections were immersed in 5 % skim milk in TBS. After incubation with each of the primary antibodies at 4 °C overnight, the sections were washed with TBS. The sections were then incubated with corresponding secondary antibodies at 37 °C for 1 h, mounted with Vectashield (H-1500, Vector Laboratories, Burlingame, CA), and examined under a Leica DMI 3000B fluorescence microscope (Leica Microsystems, Tokyo, Japan) or a Carl Zeiss LSM700 Confocal Laser Scanning Microscopy (Carl Zeiss, Tokyo, Japan). Primary antibodies used were as follows: mouse anti-hyperphosphorylated-tau Ser202/Thr205 (clone AT8, 1:100, Thermo Scientific), rabbit anti-MAP2 (1:1000, Millipore), rabbit anti-GFAP (1:400, Dako), rabbit anti-Olig2 (1:200, Millipore), mouse anti-RAB9 (clone Mab9, 1:100, LSBio, Seattle, WA), and rabbit anti-Aβ42 (1:100, IBL). Secondary antibodies used were as follows: ALEXA594-conjugated goat anti-mouse IgG (1:100, Invitrogen, Eugene, OR), ALEXA488-conjugated goat anti-rabbit IgG (1:100, Life Technologies, Eugene, OR), ALEXA594-conjugated goat anti-rabbit IgG (1:100, Life Technologies), and ALEXA488-conjugated goat anti-mouse IgG (1:100, Invitrogen).

### Transmission electron microscopy

Formalin fixed hippocampal tissues with NFTs (confirmed by Gallyas-Braak method) were cut into 1-mm cubes and then post-fixed with 2 % osmium oxide in phosphate buffer (100 mM, pH 7.2) at 4 °C for 1 h. After washed in phosphate buffer, the tissues were dehydrated in a graded series of ethanol, displaced by propylene oxide and then embedded in Spurr resin (Spurr Low Viscosity Embedding Kit, Polysciences, Warrington, PA). Ultrathin sections (70-nm-thick) were stained with 4 % uranyl acetate in distilled water and Reynolds’ lead citrate, and then examined with a JEM-1010 transmission electron microscope (JEOL, Tokyo, Japan).

### Protein extraction

For tau extraction, hippocampal tissues were homogenized in four volumes of TBS containing a protease inhibitor cocktail (cOmplete Mini, Roche, Mannheim, Germany) and a phosphatase inhibitor cocktail (PhosSTOP, Roche) and fractioned by three-step ultracentrifugation including TBS, sarkosyl (sodium N-dodecanoylsarcosinate) and guanidine hydrochloride (GuHCl) extraction, essentially as described previously [21]. For the dephosphorelation assay, phosphatase inhibitor cocktail was omitted. In brief, the homogenates were centrifuged at 125,000 × *g* at 4 °C for 1 h, and the supernatants were harvested as TBS-soluble fractions. The precipitates were dissolved by sonication in four volumes of 1 % sarkosyl in TBS containing the protease inhibitor cocktail and the phosphatase inhibitor cocktail, and then the solutions were incubated at room temperature (RT) for 1 h. After centrifugation at 125,000 × *g* at RT for 15 min, the supernatants were removed. The sarkosyl-insoluble precipitates were then dissolved by sonication in two volumes of 4 mol/L GuHCl and incubated at RT for 1 h. After a second centrifugation at 125,000 × *g* at RT for 15 min, the supernatants were harvested, and the solvent (4 mol/L GuHCl) was exchanged with TBS containing the protease inhibitor cocktail and the phosphatase inhibitor cocktail using Amicon Ultra 10 K filter devices (Millipore).

For Aβ extraction, hippocampal and parietal cortex tissues were homogenized in four volumes of TBS containing the protease inhibitor cocktail and fractioned by three-step ultracentrifugation including TBS, SDS, and FA extraction. In brief, the homogenates were centrifuged at 100,000 × *g* at 4 °C for 1 h, and the supernatants were harvested as TBS fractions. The precipitates were dissolved in four volumes of 2 % SDS in TBS containing the protease inhibitor cocktail, centrifuged at 100,000 × *g* at RT for 1 h, and the supernatants were harvested as SDS fractions. The precipitates were finally dissolved in 70 % FA in water. After centrifugation at 100,000 × *g* at RT for 1 h, the supernatants were harvested as FA fractions. The TBS and SDS fractions were diluted 10- and 20-fold, respectively, in TBS containing the protease inhibitor cocktail, and the FA fractions were neutralized by 1:10 dilution into 1 M Tris solution, pH 11. The protein concentrations of the resultant solutions were determined by the BCA protein assay (Thermo Scientific).

### Western blotting

For tau analysis, extracts of the hippocampus were incubated with alkaline phosphatase mix (500 mM Tris–HCl pH 9.0, 500 mM MgCl_2_, 0.1 M DTT (Invitrogen), 10,000U/ml calf intestinal alkaline phosphatase (New England Biolabs, Ipswich, MA)) at 37 °C overnight for dephosphorylation. Subsequently, aliquots (5 μg protein) were electrophoresed on 4-12 % Bolt Bis-Tris Plus gel (Thermo Fisher Scientific, Waltham, MA) and transferred to 0.45-μm PVDF membranes (Millipore). Nonspecific binding was blocked with 5 % skim milk in TBS containing Tween 20 (TBS-T, 20 mM Tris–HCl buffer, pH 7.0, containing 50 mM NaCl and 0.1 % Tween 20) for 30 min. The following primary antibodies were used: mouse anti-tau (clone TAU-5, 1:1000, Life Technologies), RD3 (clone 8E6/C11, 1:1000, Millipore), and RD4 (clone 1E1/A6, 1:1000, Millipore). Alkaline phosphatase-conjugated anti-mouse IgG was then applied. The blotting signals were visualized with 5-bromo-4-chloro-3'-indolylphosphatase p-toluidine salt/nitro-blue tetrazolium chloride (BCIP/NBT) and imaged with an Image Quant LAS 4000 mini bio-molecular imager (GE Healthcare Bio-Sciences AB, Uppsala, Sweden). For Aβ analysis, aliquots (50 μg protein) of the TBS and SDS fractions were electrophoresed on 4-12 % Nupage Bis-Tris polyacrylamide gels (Life Technologies) and transferred to 0.45-μm PVDF membranes (Millipore). Nonspecific binding was blocked with 5 % skim milk in TBS containing Tween 20 for 30 min. Mouse anti-Aβ antibody (clone 6E10, 1:3000), mouse anti-Aβ antibody (clone 82E1, 1 μg/ml, IBL) and rabbit anti-ApoE antibody (A299, 5 μg/ml, IBL) were used as the primary antibody. Horseradish peroxidase-conjugated secondary antibody (1:5000, Dako) was then applied. The blotting signals were visualized with the chemiluminescence ECL Select Western Blotting Detection Kit (GE Healthcare Bio-Sciences AB) and imaged with an Image Quant LAS 4000 mini bio-molecular imager (GE Healthcare).

### Dot blot

For dot blot immunoanalysis, aliquots (50 μg protein) of the TBS, SDS, and FA fractions were dotted onto 0.45-μm PVDF membranes (Millipore) in a dot-blot apparatus (Bio-Rad). The blots were probed with a polyclonal antibody A11 specific for amyloid oligomers (1:1000, Biosource, Camarillo, CA) [35], followed by alkaline phosphatase–conjugated goat anti-rabbit antibody (1:5000, Thermo Scientific), and visualized with the BCIP/NBT detection system (Wako Chemicals, Osaka, Japan).

### ELISA

To quantify the amount of Aβ oligomers in the extracts from cat brains, we used our originally developed BAN50 single-antibody sandwich ELISA that is specific for high molecular weight Aβ oligomers (10- to 20-mers) in quadruplicate [16]. The buffers and assay procedures were similar to those described previously [34]. As a standard for inter-plate calibration, we used a ‘multiple antigenic’ peptide (MAP). MAP is a synthetic peptide that consists of 16 copies of epitope peptide (corresponding to Aβ1-10) recognized by anti-Aβ monoclonal antibody (BAN50) and a single lysine core to which the epitope peptides are linked [34]. The SuperSignal ELISA Femto Maximum Sensitivity Substrate (Thermo Scientific) and a luminometer (SpectraMaxL, Molecular Devices, Osaka, Japan) were used for signal detection.

## Results

### Aβ pathology in the cat brain

**Fig. 1:**
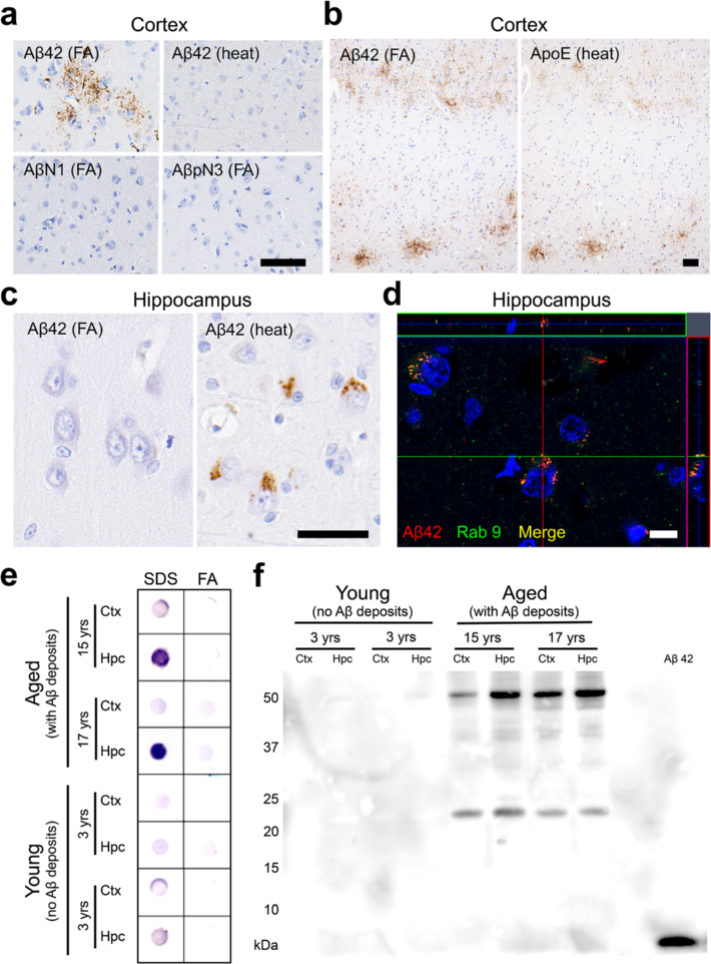
Aβ deposits in cat brains. **a** Aβ42 aggregates are detected in the parenchyma of the cerebral cortex with anti-Aβ42 antibody (12 F4) on formic acid (FA)-pretreated sections but not on heat-pretreated sections. These aggregates are not detected with antibodies against the N-terminus of human Aβ (AβN1 and AβpN3). **b** Aβ42 aggregates in the cerebral cortex colocalized with ApoE. **c** Heat pretreatment revealed intracellular Aβ42 aggregates in the pyramidal cells of the hippocampus but not in the cortex. **d** Some of the intracellular Aβ42 (red) aggregates colocalized with Rab9 (green). Black bars = 50 μm, white bar = 10 μm. **e** Dot blot analysis of SDS fractions and FA fractions of cortex (Ctx) and hippocampus (Hpc) of young cats and aged cats. Aβ oligomers were detected with A11 antibody, predominantly in the SDS fraction from the hippocampus of aged cats. **f** Western blotting analysis of the SDS fraction of the Ctx and Hpc of young cats and aged cats. Two distinct bands were detected with anti-Aβ antibody 6E10 in the brains of aged cats: approximately 24 kDa and 54 kDa, indicating Aβ hexamers and dodecamers, respectively

In the present study, we investigated domestic cats at various ages for AD pathologies. By immunohistochemical analysis with anti-Aβ42 antibody (12 F4), we found that domestic cats older than 8 years displayed Aβ deposits (Table 1). There were cases with Aβ deposits that were not associated with tau immunoreactivity but there were no case with tau positive tangles in the absence of Aβ deposits. On formic acid (FA)-pretreated sections, extracellular small granular Aβ aggregates were observed in the neuropil throughout the cerebral cortex but rarely in the hippocampus (Fig. 1a, Additional file 1: Figure S1a). These parenchymal Aβ deposits in cat brains had no central core as seen in mature plaques of human AD and Tg mouse models of AD, and were not visualized by silver staining or Congo red staining. Also, vascular Aβ deposition and neuritic alterations were absent. As it has been previously demonstrated [11, 26], full-length Aβ1-42 aggregates in the cat brains were not stained with Aβ N-terminal antibody anti-AβN1 (Fig. 1a). In addition, unlike typical SPs in human, monkey and dog brains [7, 15], Aβ aggregates in the cat brains were not stained with antibody against N-terminally truncated Aβ (AβpN3). ApoE protein colocalized with the small granular Aβ aggregates in the cerebral cortex (Fig. 1b). In contrast to the staining pattern of Aβ in the cerebral cortex, on heat-pretreated sections, intracellular accumulation of Aβ was detected predominantly in the pyramidal cells of the hippocampal CA1 to CA3 region but rarely in the cerebral cortex (Fig. 1c, Additional file 1: Figure S1b). ApoE immunopositivity was not detected in this area. These intracellular Aβs co-localized with Rab9 under confocal laser scanning microscopy, indicating their localization in late endosomes (Fig. 1d). The FA-vulnerable intracellular Aβs in the hippocampus always coincided with FA-resistant parenchymal Aβ deposits in the cerebral cortex. The different states of Aβ aggregation between the hippocampus and cerebral cortex may be due to the different neuronal cell types and/or different environments surrounding neurons in these regions. Dot blot analysis using amyloid oligomer-specific A11 antibody revealed the existence of Aβ oligomers in SDS-soluble fractions, but not in FA-soluble fractions, prepared from hippocampal tissues of aged cats (Fig. 1e). Considering the vulnerability to FA treatment, these Aβ oligomers were presumably derived from the intracellularly accumulated Aβs observed in immunohistochemistry. Western blotting analysis using anti-Aβ antibody 6E10 indicated two distinct bands with molecular sizes of approximately 24 kDa and 54 kDa, which corresponded to Aβ hexamers and dodecamers, respectively, in the SDS-soluble fractions (Fig. 1f). The band corresponding to Aβ dodecamer was detected by anti-Aβ antibody 82E1, an antibody that does not react with APP (Additional file 1: Figure S2a). Neither of the bands corresponding to Aβ hexamers or dodecamers was detected with anti-ApoE antibody, indicating that these oligomers are not bound with ApoE (Additional file 1: Figure S2b). The presence of Aβ oligomers in the SDS-soluble fractions was also confirmed by enzyme-linked immunosorbent assay (ELISA) specific for high molecular weight Aβ oligomers including Aβ dodecamer (Additional file 1: Figure S1c).

### Spatial and temporal expression of tau isoforms in the cat brain

**Fig. 2:**
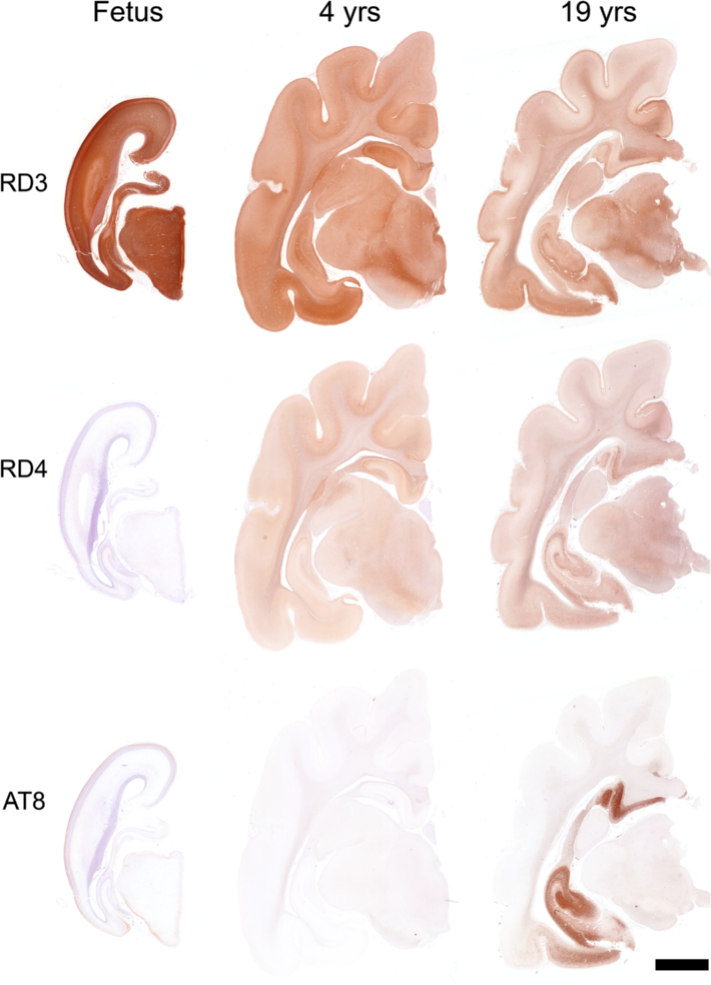
Immunohistochemical analysis of tau isoforms and their phosphorylation status in cat brains. Immunohistochemistry for 3-repeat tau (RD3), 4-repeat tau (RD4), and hyperphosphorylated-tau (AT8) in the developing cat (Fetus, case No. 1 shown in Table 1), adult cat (4 years (yrs) old, case No. 7), and aged cat (19 yrs old, case No. 22) brains. Only the 3-repeat tau isoform is expressed in the fetal cat brain, whereas both 3-repeat and 4-repeat tau isoforms are expressed in the developed cat brains. Abundant AT8-positive hyperphosphorylated tau aggregates are observed in the hippocampus and entorhinal cortex of the aged cat brain. Bar = 5 mm

**Fig. 3:**
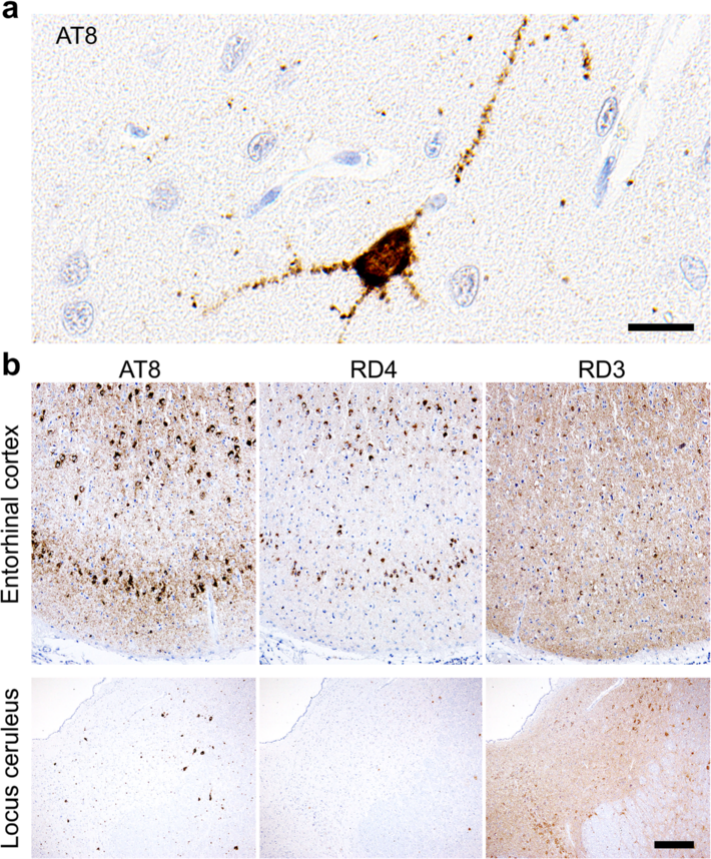
Hyperphosphorylated tau accumulation in the entorhinal cortex and locus ceruleus of cat brains. **a** Immunohistochemistry of the entorhinal cortex of a cat with mild hyperphosphorylated tau accumulation (15-year-old, case No. 13) for hyperphosphorylated-tau (AT8). The neuronal soma and dendrites are positively stained for hyperphosphorylated tau. Bar = 20 μm. **b** Immunohistochemistry of the entorhinal cortex and locus ceruleus of a cat with severe hyperphosphorylated tau accumulation (14-year-old, case No. 12) for AT8, 3-repeat tau (RD3), and 4-repeat tau (RD4). AT8-positive aggregates are also positively stained for 3-repeat tau and 4-repeat tau on consecutive sections. Bar = 300 μm

**Fig. 4:**
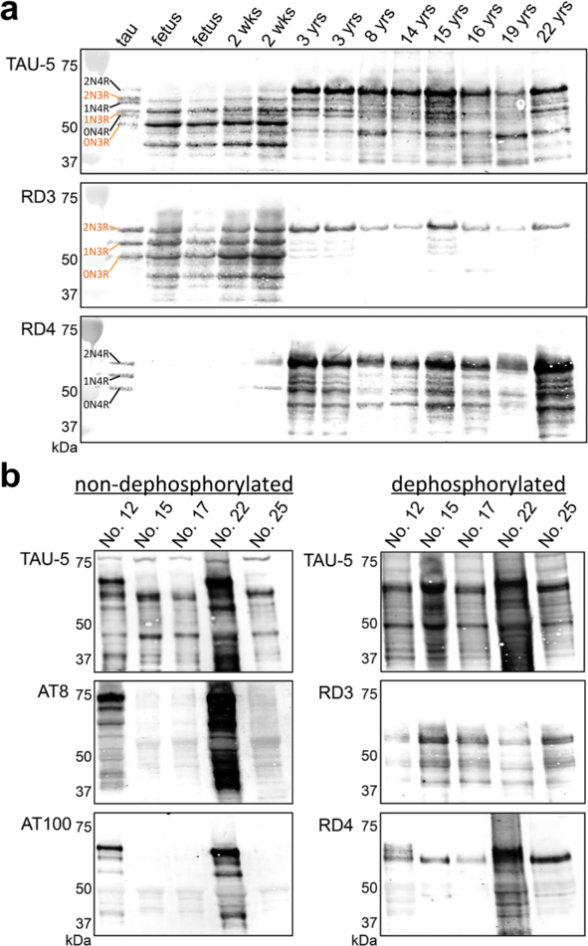
Western blotting analysis of tau isoforms and their phosphorylation status in cat brains. **a** Western blotting of TBS-soluble fractions obtained from the hippocampus of various ages and treated with alkaline phosphatase (AP). The left lane (tau) shows the six isoforms of human tau (recombinantly produced): three 3-repeat tau isoforms (2N3R, 1N3R, 0N3R) and three 4-repeat tau isoforms (2N4R, 1N4R, 0N4R). In the fetal brain, only the 3-repeat tau isoforms are expressed. In the adult cat brains, all six isoforms are detected using anti-tau antibody (TAU-5) and also 3-repeat tau (RD3), and 4-repeat tau (RD4) antibodies. **b** Western blotting of sarkosyl-insoluble guanidine HCl-soluble fractions obtained from the hippocampus of aged cat brains without AP treatment (left) and with AP treatment (right). In cat hippocampi that were immunohistochemically positive for hyperphosphorylated tau (cases No. 12 and 22), AT8- and AT100-positive tau proteins are detected. In these cats, the smear-like band pattern resolved into clear lower molecular weight bands consisting of both 3-repeat and 4-repeat tau isoforms after dephosphorylation treatment

We next examined tau pathology in cat brains. Initially, we studied the expression pattern of tau isoforms among different ages. In immunohistochemistry with RD3 and RD4 antibodies specific to 3-repeat (3R) and 4-repeat (4R) tau, respectively, fetal cats were shown to express only 3R tau throughout the cerebrum (Fig. 2). The hippocampal pyramidal cells began to express 4R tau at 2 weeks postnatal age (Additional file 1: Figure S3a). In adult brains (3- to 22-year-old), both 3R and 4R tau were detected. We next investigated tau hyperphosphorylation by immunohistochemistry with AT8 (anti-pSer202/pThr205-tau) and AT100 (anti-pThr212/pSer214-tau) antibodies. All of the cats without Aβ deposits were negative for hyperphosphorylated tau, except in fetal cats AT8-positive staining was detected in the surface layer of the cerebrum (Table 1, Additional file 1: Figure S3b). In some of the aged cats with Aβ deposits (over 14 years old), AT8-positive staining was detected within neurons (Table 1). In mild cases, a few AT8-positive cells were observed in the entorhinal cortex (Fig. 3a), and in more severe cases, numerous AT8-positive cells were detected throughout the entorhinal cortex, hippocampus and also mildly in the cerebral cortex (Figs. 2, 3b, Table 1). Also, a few AT8-positive cells were observed in the locus ceruleus of aged cats (Fig. 3b). We noticed that the cerebrums were atrophied in aged cats compared to those in young cats (Fig. 2). The age-dependent change in tau isoform expression was confirmed with western blotting with anti-tau (TAU-5), RD3, and RD4 antibodies of TBS-soluble fractions prepared from the cerebrums (Fig. 4a). Western blotting analysis with AT8 and AT100 antibodies indicated that sarkosyl-insoluble, guanidine HCl-soluble fractions prepared from hippocampal tissues of aged cats contained abundant hyperphosphorylated tau (Fig. 4b). These insoluble tau species, which showed a smear profile in western blots, were shown to consist of both 3R and 4R tau isoforms, which was proven after dephosphorylation of the samples (Fig. 4b).

### NFT in aged cat brains

**Fig. 5:**
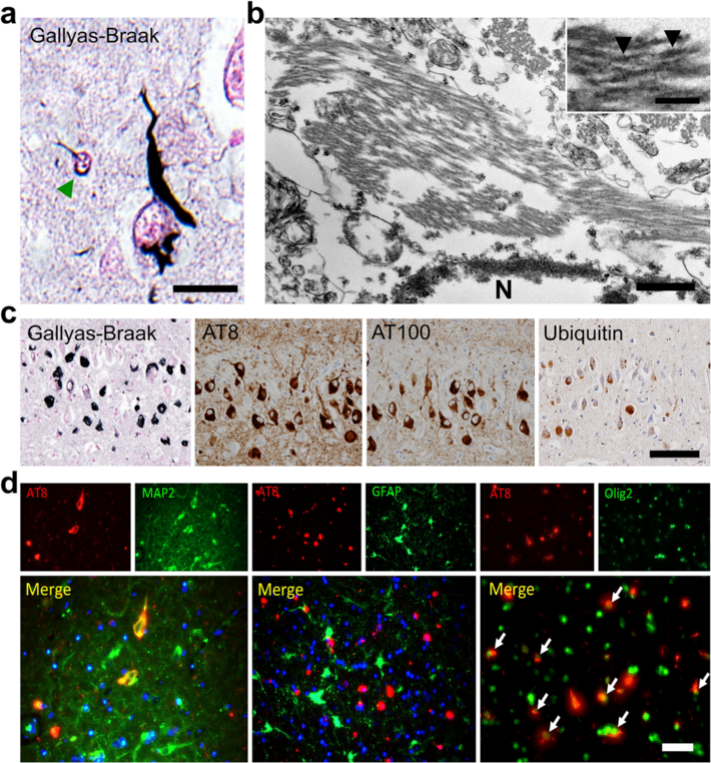
NFTs in aged cats brains. **a** Gallyas-Braak staining-positive argyrophilic aggregates are observed mainly in the neuronal soma, neurites, and also in some oligodendroglial cells (green arrowhead) in the entorhinal cortex of aged cat brain. Bar = 20 μm. **b** Transmission electron microscopy of NFT in the hippocampus. Bundles of filaments are observed in the neuronal soma either in strait form or paired twisted form. For the paired twisted form, the lengths between the constrictions (arrowheads) were 80-100 nm. Bars = 500 nm and 100 nm (inset). N: nucleus. **c** Consecutive sections of hippocampus show AT8-, AT100-, and ubiquitin-immunopositivity for NFTs. Bar = 100 μm. **d** AT8-positive (red) hyperphosphorylated tau is observed in MAP2-positive (green) neurons (left) and Olig2-positive (green) oligodendrocytes (right, white arrows), but not in GFAP-positive (green) astrocytes (middle). Bar = 50 μm

The presence of hyperphosphorylated tau in insoluble brain fractions implies that these tau proteins may form NFTs. Thus, we examined NFT formation in aged cats by Gallyas-Braak silver staining and confirmed silver positive fibrillar aggregates in the neuronal somata and in neurites (Fig. 5a). By electron microscopy, bundles of filaments were observed in the neuronal somata and neurites (Fig. 5b). These filaments had widths of 15-25 nm and showed either straight pattern or paired twisted pattern. In the twisted area, lengths between the constrictions were 80-100 nm. These ultrastructural findings were comparable to those of NFTs in AD [2], although the lengths between the constrictions of the paired twisted filaments tend to be a little longer than in AD. Abundant NFTs were found in the hippocampus (Fig. 5c), but rarely in the cerebral cortex (Fig. 2), of aged cats. Notably, this distribution of NFTs in aged cat brains corresponded to that of intracellular Aβ oligomers. In consecutive sections, NFTs were co-localized with hyperphosphorylated tau (AT8 and AT100) and ubiquitin (Fig. 5c). Regarding the affected cell types, hyperphosphorylated tau was detected mainly in neurons (MAP2-positive) and in some oligodendrocytes (Olig2-positive), but not in astrocytes (GFAP-positive) (Fig. 5d). Aggregates in the oligodendrocytes were also detected by Gallyas-Braak staining (Fig. 5a). The absence of astrocytic tau inclusion bodies, such as astrocytic plaques or tuft-shaped astrocytes, indicates that the hyperphosphorylated tau aggregates found in feline brains differ from those associated with corticobasal degeneration and progressive supranuclear palsy [28].

### Hippocampal neuronal loss in cats with NFT

**Fig. 6:**
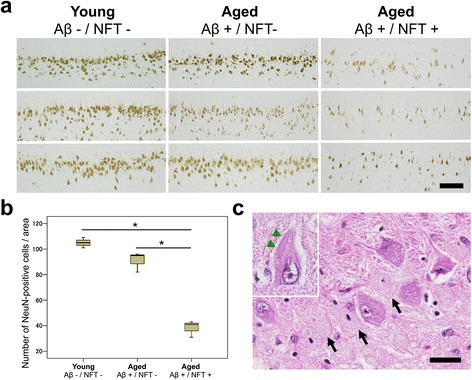
Hippocampal neuronal loss in cats with NFTs. **a** Immunohistochemistry for NeuN in young cats (Aβ–/NFT–, *n* = 3, mean age 3.7 years old), aged cats with Aβ deposits but no NFTs in the hippocampus (Aβ+/NFT–, *n* = 3, mean age 18 years old), and aged cats with Aβ deposits and NFTs (Aβ+/NFT+, *n* = 3, mean age 17.6 years old). Bar = 100 μm. **b** The number of NeuN-positive pyramidal cells shown in (**a**). The number of hippocampal neurons is significantly decreased in aged cats with Aβ deposits and NFTs (Aβ+/NFT+) compared to young cats (Aβ–/NFT–) and aged cats with Aβ deposits but no NFTs in the hippocampus (Aβ+/NFT–). *P < 0.001. **c** Ghost tangles are observed on an HE-stained section (arrows). In some of the cells, inclusions (composed of hyperphosphorylated tau, confirmed on consecutive sections) were observed (inset, arrowheads). Bar = 25 μm

Although neuronal loss in the entorhinal cortex and hippocampus is considered to be an early event in AD [22, 59], it has not been detected in combination with Aβ and tau pathologies in non-human species [50, 58]. Because hippocampal neurodegeneration is important in the manifestation of AD [30], we assessed neuronal loss in the hippocampus of the cats. The number of NeuN-positive cells in the hippocampal CA1 region were compared among three groups: young cats with neither Aβ deposits nor NFTs (Aβ-/NFT-, *n* = 3, mean age 3.7 years old), aged cats with only cerebral Aβ deposits but no hippocampal NFTs (Aβ+/NFT-, *n* = 3, mean age 18.0 years old), and aged cats with both cerebral Aβ deposits and hippocampal NFTs (Aβ+/NFT+, *n* = 3, mean age 17.6 years old). In the Aβ+/NFT- group, NeuN-positive cells were slightly but not significantly decreased compared to those in the Aβ-/NFT- group (*P* = 0.076, Fig. 6a, b), whereas the Aβ+/NFT+ group showed a significant decrease in NeuN-positive cells compared to the other two groups (*P* < 0.001, Fig. 6a, b). Ghost tangles were visible on HE-stained sections suggesting the death of tangle bearing neurons (Fig. 6c, black arrows). These dead neurons were negative for NeuN and the live cells were positive for NeuN. Also, inclusions composed of hyperphosphorylated tau (AT8- and AT100-positive on consecutive sections) were occasionally observed on HE-stained sections (Fig. 6c, inset, green arrowheads).

## Discussion

In this study, we found that aged domestic cats develop not only Aβ deposits but also NFTs and neuronal loss in their brains. Though it has been shown that NFT occurs earlier than senile plaque in humans, in cats Aβ deposits (diffuse plaques in the cerebral cortex and intracellular Aβ oligomers in the hippocampus) started to occur at 8 years of age, and NFTs and neuronal loss at 14 years of age. The distribution, affected cells, tau isoforms and ultrastructure of the NFTs were comparable to those of AD. NFT formation and subsequent neuronal loss occurred in the same brain region as that of intracellular Aβ oligomer accumulation, i.e., the hippocampus. These findings have also been seen in Tg mouse models and human AD patients [1, 17, 41, 57]. The early occurrence of these pathologies makes this animal species an attractive model for studying therapeutic intervention for AD.

What factors enable domestic cats to develop full AD pathologies, particularly NFTs and neuronal loss, in its shorter life-span than humans? Current evidence suggests that Aβ and tau interact to accelerate each other’s pathology and that tau hyperphosphorylation and subsequent NFT formation are induced by pathological Aβ species, i.e., Aβ oligomers [3, 29]. Our results demonstrating that NFTs formed in the same brain region as intracellular Aβ oligomers in aged cats may imply the involvement of Aβ oligomers in the initiation (early stage) of NFT development. Furthermore, these Aβ oligomers consisted of Aβ hexamers and dodecamers (Fig. 1c), which are considered to be pathological Aβ species and to be associated with pathological tau conformers in AD [3, 4, 37]. The accumulation of Aβ oligomers in domestic cats presumably comes from its Aβ sequence, in which the 7th amino acid residue is different from that of human Aβ [5, 39] (Additional file 1: Table S1). There are two known familial AD-linked mutations in the N-terminal region of Aβ: the English mutation (H6R) and the Tottori mutation (D7N), both of which result in increased formation of Aβ oligomers [9, 43]. Also, racemization of the 7th Asp residue affects the Aβ aggregation property and inhibits its fibril formation [53]. Thus, the substitution of the 7th amino acid of human Aβ likely results in enhanced oligomerization and reduced fibrilization of Aβ, which may explain why cat Aβ abundantly accumulates into oligomeric forms.

This study demonstrated that domestic cats develop non-argyrophilic small granular Aβ aggregates in the cerebral cortex and FA-vulnerable intracellular Aβ oligomers in the hippocampus, but not argyrophilic SPs. ApoE colocalized with the non-argyrophilic Aβ aggregates in the cerebral cortex that lack immunoreactivity against anti-Aβ N-terminus antibodies. The same finding has been described with newly formed Aβ deposits in the human brain indicating that the N-terminal epitope of the Aβ is bound with ApoE [51]. We consider that the lack of SP formation is important for the early development of NFTs in cat brains. Many animal species including monkeys, dogs, bears, camels, and horses, whose Aβ sequence is identical to that of human Aβ (Additional file 1: Table S1), spontaneously develop abundant argyrophilic SPs similar to human SPs in old age, but do not develop NFTs [6, 15, 33, 40, 48, 56, 58]. It is known that the extent of intraneuronal Aβ42 labeling is inversely correlated with the progression of SP in double Tg (APP_SWE/London_ and mutant PS1 _M146L_) and triple Tg (APP_SWE_, tau_P301L_, and PS1_M146V_ knock-in) mice, suggesting that the amount of intraneuronal soluble Aβ is in equilibrium with the amount of extraneuronal insoluble fibrillar Aβ [36, 42]. Furthermore, the number of Aβ oligomers present within the brain is inversely correlated with the severity of SP in aged dogs [25]. Thus it has been proposed that SPs are formed to sequester toxic Aβ oligomers, preventing Aβ oligomer-induced pathologies including NFT formation [3]. In animal species that express human-type Aβ, SPs may be gathering toxic Aβ oligomers as “trash bins”, preventing Aβ oligomers from interacting with tau and proceeding to NFT formation [12, 38, 52] (Table 2). Uniquely among animals, humans have extended their life-span to overwhelm the buffering capacity of SPs, and Aβ oligomers that have overflowed SPs could cause tau pathologies. In cats, on the other hand, the buffering stage (SP formation) is skipped, and therefore, tau pathologies would appear soon after Aβ oligomer formation in a shorter life-span. A very similar type of Aβ to cat Aβ is known in human familial AD. Patients and Tg mice with the APP Osaka (E693Δ) mutation, which corresponds to E22Δ in the Aβ sequence, accumulate abundant Aβ oligomers within neurons without developing SPs and showed early cognitive dysfunction [54, 55]. Furthermore, when the Tg mice were crossbred with tau-Tg mice expressing both 3R and 4R human tau, the resultant double Tg mice develop NFTs [57].

**Table 2:** Comparison of AD pathology in different species [5, 19, 25, 27, 31, 33, 39, 44, 48]

Species	Life span	Aβ			Tau			Neuron loss
		Sequence vs. human	Oligomers	SP	Sequence vs. human	Isoforms^a^	NFT	
Human	80 yrs	—	Yes	Yes	—	3R + 4R (6)	Yes	Yes
Chimpanzee	60 yrs	100 %	ND	Yes	100 %	3R + 4R (6)	No	No
Dog	20 yrs	100 %	Yes	Yes	92 %	3R + 4R (4)	No	No
Cat	20 yrs	1 a.a. different	Yes	No	93 %	3R + 4R (6)	Yes	Yes
Mouse	2 yrs	3 a.a. different	No	No	89 %	4R (3)	No	No

^a^parenthesis indicate the number of total tau isoforms; yrs, years; a.a., amino acid(s); ND, no data

Besides the accumulation of Aβ oligomers, other factors may also be involved in NFT formation in domestic cats. We first assumed that the expression pattern of tau isoforms might influence NFT formation. The expression patterns of 3R and 4R tau differ among animal species and developmental stages [10, 20, 31]. In humans and mice, only 3R tau isoforms are expressed in the fetal brain. In adults, whilst both 3R and 4R tau isoforms are expressed in humans, only 4R tau isoforms are expressed in mice. As mentioned above, mice do not develop NFT, but tau Tg mouse strains that express both 3R and 4R tau isoforms do [57]. In the present study, we showed that only 3R tau isoforms are expressed in the brains of fetal cats, whereas both 3R and 4R tau isoforms were detected in the brains of adult cats. Thus, the expression of both 3R and 4R tau isoforms seems to be a prerequisite for NFT formation. However, chimpanzees, which also express both 3R and 4R tau isoforms in adult ages, do not develop NFTs except in the case of brain infarction [19, 23, 27, 44, 48]. We also considered the involvement of the tau amino acid sequence. Mouse tau shows only 83 % homology with human tau, whereas cat tau shows 93 % homology with human tau (Table 2, Additional file 1: Table S2). However, again, chimpanzees whose tau sequence is identical to that of human tau do not develop NFTs. These observations suggest that tau isoform expression and the amino acid sequence may be important for NFT formation, but the pathology does not simply depend on either of these factors.

The present study detected intracellular Aβ accumulation, NFT formation, and neuronal loss in the hippocampi of aged cats’ brains. However, no significant neuronal loss was observed in the brains of cats that were free from NFT, even when intracellular Aβ was present (Fig. 6a, b). This implies that NFTs are important for hippocampal neurodegeneration, as has been indicated in studies of human AD [30]. Some studies have suggested that the toxic tau species is oligomers, and NFTs are formed to sequester those oligomers [45]. If so, when NFTs are saturated, tau oligomers would overflow causing neurodegeneration. This may explain our observation that neuronal loss and NFTs were observed in the same brain region.

In a previous report, AT8-positivity was detected in cats with seizures [26]. In the present study, none of the cats had been reported to have seizures. Also, we performed full necropsy of all the cases and did not find lesions that would cause seizures, thus AT8-positivity and the severe neuronal loss are not related to seizure activity in cats.

## Conclusions

It has been argued that the pathology observed in Tg mice harboring mutations in their *APP*, *PSEN*, and/or *MAPT* is different from that seen in human AD, probably due to the abnormally high expression levels and/or altered aggregation properties of Aβ and tau [13, 14, 41, 46]. Non-Tg animals, such as monkeys and dogs, develop SP composed of human-type Aβ as they age, but these animals do not experience NFT-induced neurodegeneration. Here, we demonstrated that domestic cats spontaneously develop Aβ deposition, NFT formation, and neuronal loss, during their shorter life-span (about 20 years) than that of humans. Based on these pathological features of aged cat brains, we propose that domestic cats could be a valuable natural animal model of human AD, as aged cats display Aβ and tau pathologies earlier than humans, and thus, would be useful for investigating the pathogenesis of and possible treatments for the disease.

## Additional file

Additional file 1:
**Figure S1.** Aβ deposition in cat brains. (**a**) Immunohistochemistry of the cerebrum of a 17-year-old cat (case No. 20) for Aβ42 with FA pretreatment. Aβ42 aggregates are observed in the cerebral cortex but not in the hippocampus by immunohistochemistry with FA pretreatment. Higher magnification of the parietal lobe (right). Bar = 100 μm. (**b**) Negative control (without primary antibody) of Fig. 1c. No staining is detected in the cytoplasm. (**c**) ELISA for high molecular weight Aβ oligomers. Higher amounts of Aβ oligomers were seen in the brains of aged cats (15-year-old, case No. 15; 17-year-old, case No. 20) compared to the brains of young cats (3-year-old, case No. 5; 3-year-old, case No. 6). The ratio of MAP level per unit of Aβ concentration varies depending on the sizes of the Aβ oligomers. One pM of the MAP can be estimated to yield the same signal as 1.56 pM (for 20-mer) to 5.0 pM (for 100-mer) of Aβ42 oligomers [33]. Ctx, cortex; Hpc, hippocampus. **Figure S2** Western blotting analysis of the SDS fraction of the cortex (Ctx) and hippocampus (Hpc) of young cats and aged cats. (**a**) The band corresponding to Aβ dodecamer is detected by anti-Aβ antibody 82E1. (**b**) Aβ oligomers that are demonstrated in Fig. 1f are not detected by anti-ApoE antibody A299. **Figure S3** Expression of tau isoforms in the developing cat brain. (**a**) Immunohistochemistry of the hippocampus CA1 region of a fetus (case No. 1), a 2-week-old cat (case No. 3), and a 4-year-old cat (case No. 7) for 3-repeat tau (RD3), 4-repeat tau (RD4), and hyperphosphorylated tau (AT8). Only the 3-repeat tau isoform is expressed in the fetal hippocampus. The hippocampal pyramidal cells begin to express 4-repeat tau in the 2-week-old cat (arrows). Both 3-repeat and 4-repeat tau isoforms are expressed in the hippocampus of adult cat brain. Bar = 50 μm. (**b**) Immunohistochemistry of the cerebral cortex of a fetal cat for hyperphosphorylated tau (AT8 and AT100). The surface layer of the fetal cerebral cortex is positive for AT8 and negative for AT100. Bar = 100 μm. **Table S1** Aβ protein amino acid sequences of different animal species. **Table S2** Tau protein amino acid sequences of different animal species. (PDF 10834 kb)
