# Supplementary material for: The domestic cat as a natural animal model of Alzheimer’s disease
Source: Acta Neuropathol Commun. 2015 Dec 10;3:78. doi: 10.1186/s40478-015-0258-3 (PMC4674944; doi:10.1186/s40478-015-0258-3)
Supplement: Additional file 1: — Figure S1. Aβ deposition in cat brains. (a) Immunohistochemistry of the cerebrum of a 17-year-old cat (case No. 20) for Aβ42 with FA pretreatment. Aβ42 aggregates are observed in the cerebral cortex but not in the hippocampus by immunohistochemistry with FA pretreatment. Higher magnification of the parietal lobe (right). Bar = 100 μm. (b) Negative control (without primary antibody) of Fig. 1c. No staining is detected in the cytoplasm. (c) ELISA for high molecular weight Aβ oligomers. Higher amounts of Aβ oligomers were seen in the brains of aged cats (15-year-old, case No. 15; 17-year-old, case No. 20) compared to the brains of young cats (3-year-old, case No. 5; 3-year-old, case No. 6). The ratio of MAP level per unit of Aβ concentration varies depending on the sizes of the Aβ oligomers. One pM of the MAP can be estimated to yield the same signal as 1.56 pM (for 20-mer) to 5.0 pM (for 100-mer) of Aβ42 oligomers [33]. Ctx, cortex; Hpc, hippocampus. Figure S2 Western blotting analysis of the SDS fraction of the cortex (Ctx) and hippocampus (Hpc) of young cats and aged cats. (a) The band corresponding to Aβ dodecamer is detected by anti-Aβ antibody 82E1. (b) Aβ oligomers that are demonstrated in Fig. 1f are not detected by anti-ApoE antibody A299. Figure S3 Expression of tau isoforms in the developing cat brain. (a) Immunohistochemistry of the hippocampus CA1 region of a fetus (case No. 1), a 2-week-old cat (case No. 3), and a 4-year-old cat (case No. 7) for 3-repeat tau (RD3), 4-repeat tau (RD4), and hyperphosphorylated tau (AT8). Only the 3-repeat tau isoform is expressed in the fetal hippocampus. The hippocampal pyramidal cells begin to express 4-repeat tau in the 2-week-old cat (arrows). Both 3-repeat and 4-repeat tau isoforms are expressed in the hippocampus of adult cat brain. Bar = 50 μm. (b) Immunohistochemistry of the cerebral cortex of a fetal cat for hyperphosphorylated tau (AT8 and AT100). The surface layer of the fetal cerebral cortex is positive for AT8 [file 40478_2015_258_MOESM1_ESM.pdf]

Supplementary material

The domestic cat as a natural animal model of Alzheimer's disease

Chambers JK et al.

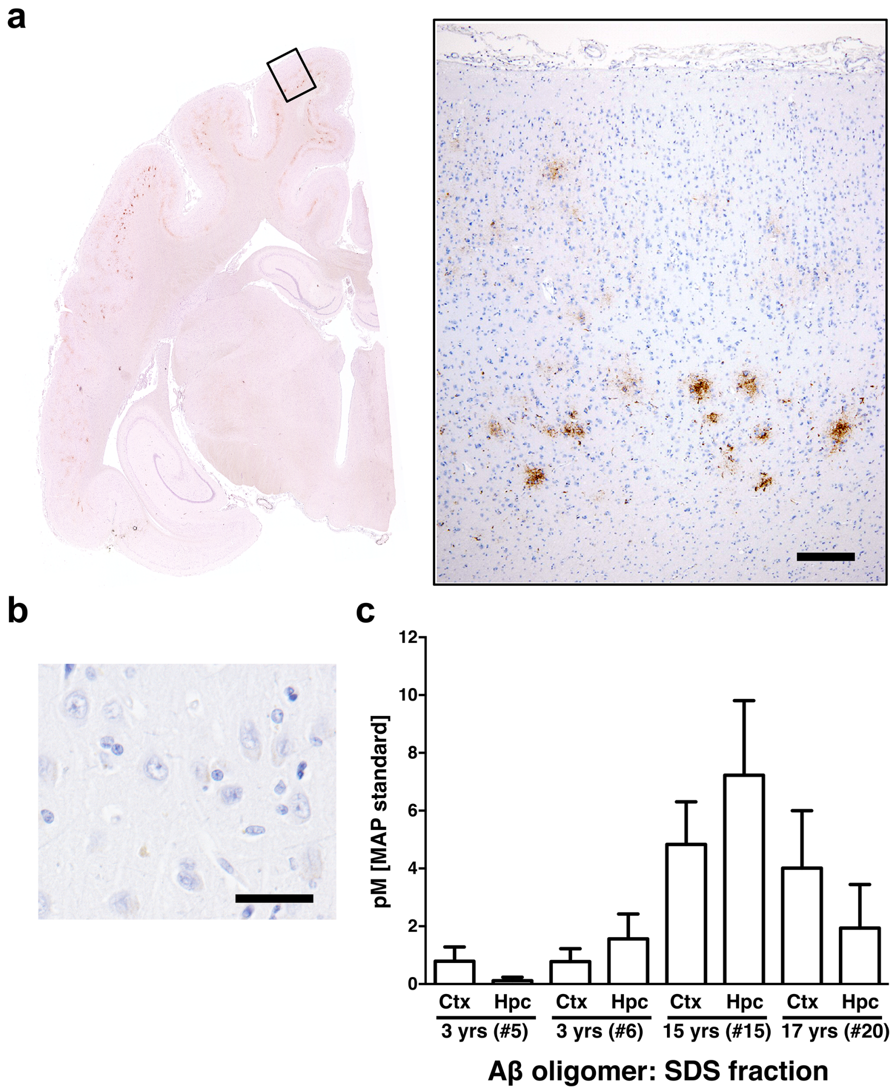

Supplementary Fig. 1 Aβ deposition in cat brains. (a) Immunohistochemistry of the cerebrum of a 17-year-old cat (case No. 20) for Aβ42 with FA pretreatment. Aβ42 aggregates are observed in the cerebral cortex but not in the hippocampus by immunohistochemistry with FA pretreatment. Higher magnification of the parietal lobe (right). Bar = 100 μm. (b) Negative control (without primary antibody) of Fig. 1c. No staining is detected in the cytoplasm. (c) ELISA for high molecular weight Aβ oligomers. Higher amounts of Aβ oligomers were seen in the brains of aged cats (15-year-old, case No. 15; 17-year-old, case No. 20) compared to the brains of young cats (3-year-old, case No. 5; 3-year-old, case No. 6). The ratio of MAP level per unit of Aβ concentration varies depending on the sizes of the Aβ oligomers. One pM of the MAP can be estimated to yield the same signal as 1.56 pM (for 20-mer) to 5.0 pM (for 100-mer) of Aβ42 oligomers [33]. Ctx, cortex; Hpc, hippocampus.

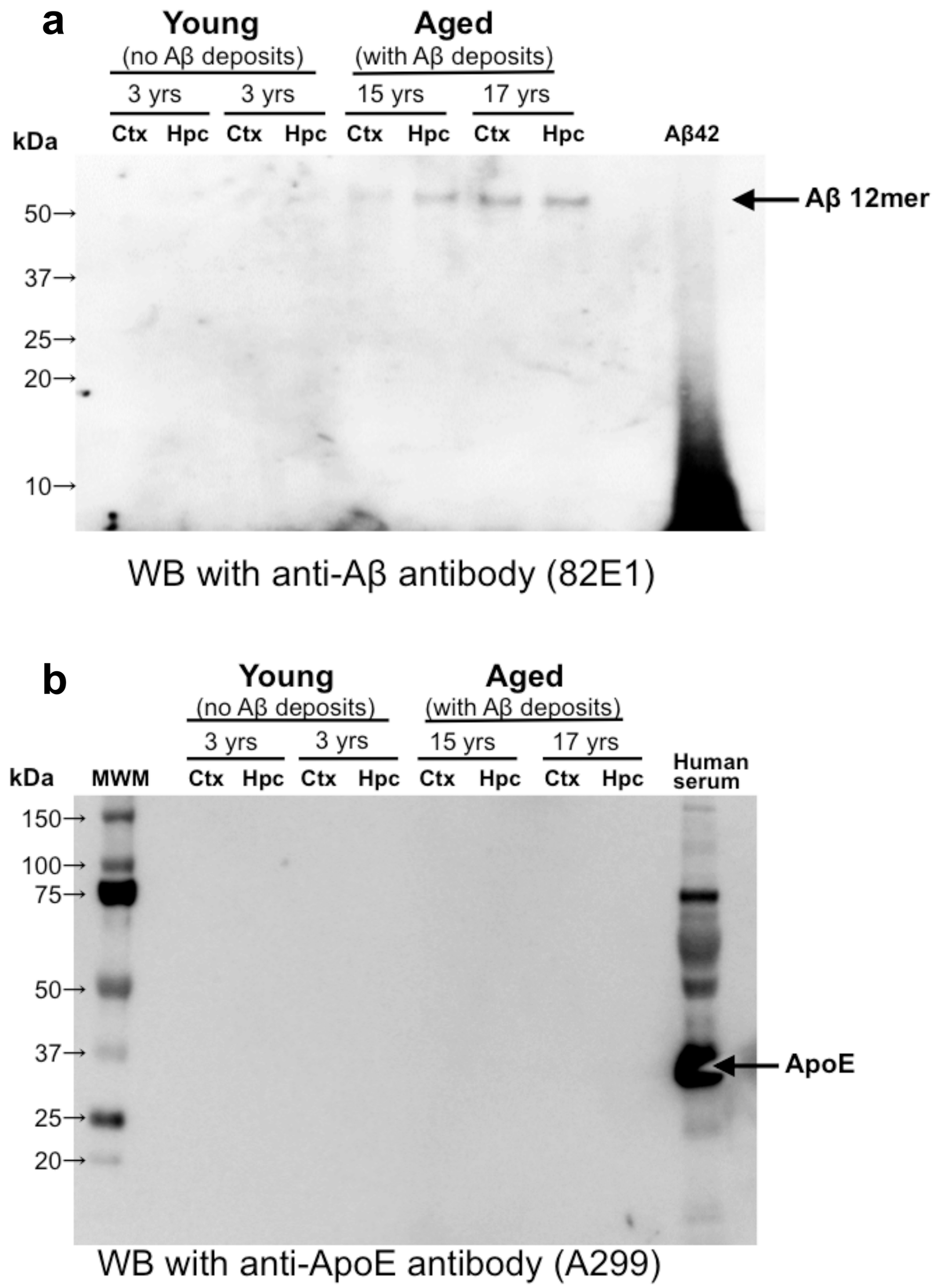

Supplementary Fig. 2 Western blotting analysis of the SDS fraction of the cortex (Ctx) and hippocampus (Hpc) of young cats and aged cats. (a) The band corresponding to Aβ dodecamer is detected by anti-Aβ antibody 82E1. (b) Aβ oligomers that are demonstrated in Fig. 1f are not detected by anti-ApoE antibody A299.

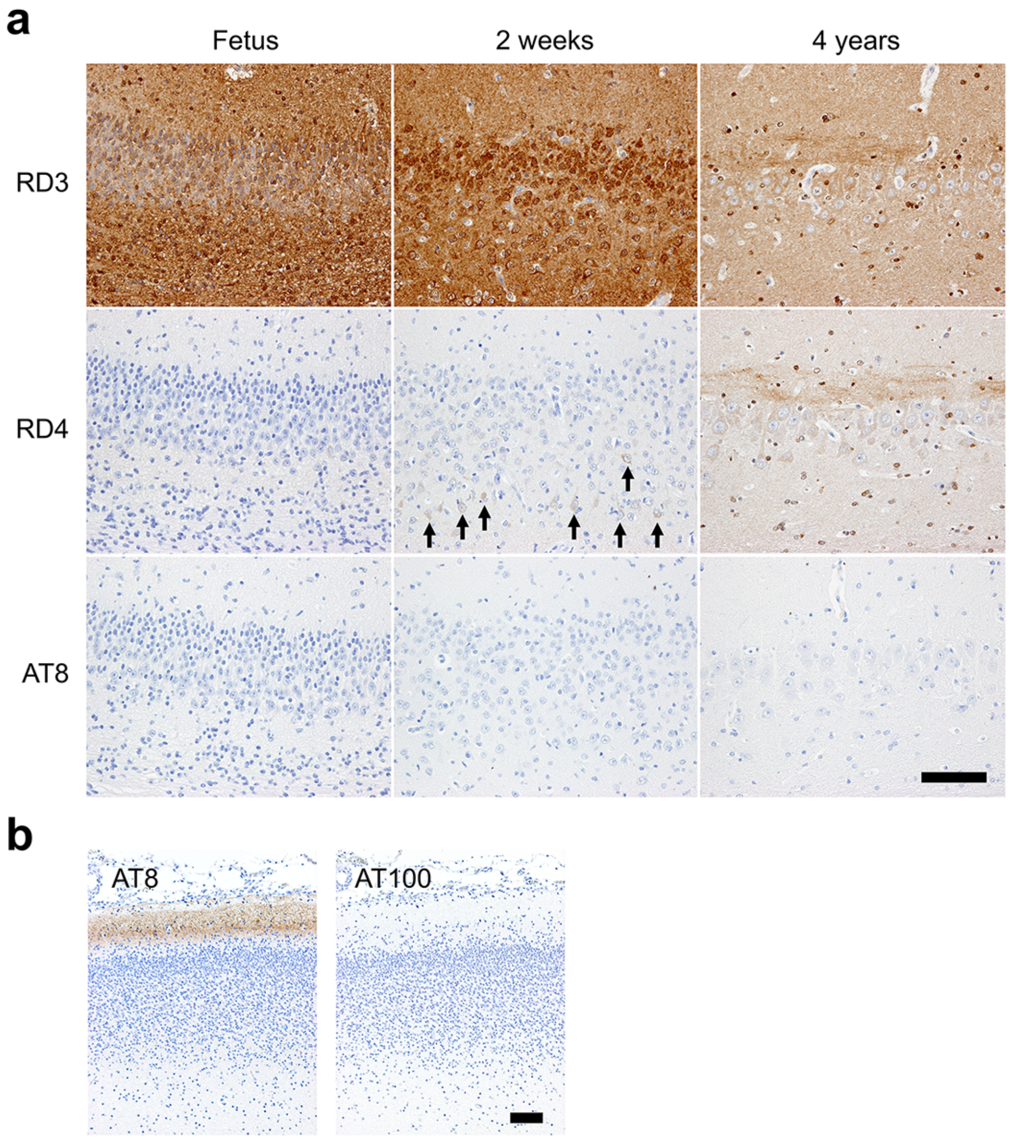

Supplementary Fig. 3 Expression of tau isoforms in the developing cat brain. (a) Immunohistochemistry of the hippocampus CA1 region of a fetus (case No. 1), a 2-week-old cat (case No. 3), and a 4-year-old cat (case No. 7) for 3-repeat tau (RD3), 4-repeat tau (RD4), and hyperphosphorylated tau (AT8). Only the 3-repeat tau isoform is expressed in the fetal hippocampus. The hippocampal pyramidal cells begin to express 4-repeat tau in the 2-week-old cat (arrows). Both 3-repeat and 4-repeat tau isoforms are expressed in the hippocampus of adult cat brain. Bar = 50 μm. (b) Immunohistochemistry of the cerebral cortex of a fetal cat for hyperphosphorylated tau (AT8 and AT100). The surface layer of the fetal cerebral cortex is positive for AT8 and negative for AT100. Bar = 100 μm.

Supplementary Table1 Aβ protein amino acid sequences of different animal species.

	Species [species, sequence ID]		Amino acid sequence	
Human-type Aβ	Human [*Homo sapiens*, NP_000475.1]	1	D A E F R H D S G Y E V H H Q K L V F F A E D V G S N K G A I I G L M V G G V V I A	42
	Chimpanzee [*Pan troglodytes*, NP_001013036.1]	1	D A E F R H D S G Y E V H H Q K L V F F A E D V G S N K G A I I G L M V G G V V I A	42
	Cynomolgus monkey [*Macaca fascicularis*, XP_005548940.1]	1	D A E F R H D S G Y E V H H Q K L V F F A E D V G S N K G A I I G L M V G G V V I A	42
	Dog [*Canis familiaris*, NP_001006601.2]	1	D A E F R H D S G Y E V H H Q K L V F F A E D V G S N K G A I I G L M V G G V V I A	42
	Polar bear [*Ursus maritimus*, XP_008699989.1]	1	D A E F R H D S G Y E V H H Q K L V F F A E D V G S N K G A I I G L M V G G V V I A	42
	Camel [*Camelus bactrianus*, XP_010954929.1]	1	D A E F R H D S G Y E V H H Q K L V F F A E D V G S N K G A I I G L M V G G V V I A	42
	Horse [*Equus caballus*, XP_003364220.1]	1	D A E F R H D S G Y E V H H Q K L V F F A E D V G S N K G A I I G L M V G G V V I A	42
Felid-type Aβ	Cat [*Felis catus*, XP_006936005.1]	1	D A E F R H E S G Y E V H H Q K L V F F A E D V G S N K G A I I G L M V G G V V I A	42
Rodent-type Aβ	Mouse [*Mus musculus*, NP_001185752.1]	1	D A E F G H D S G F E V R H Q K L V F F A E D V G S N K G A I I G L M V G G V V I A	42
	Rat [*Rattus norvegicus*, NP_062161.1]	1	D A E F G H D S G F E V R H Q K L V F F A E D V G S N K G A I I G L M V G G V V I A	42

Supplementary Table 2 Tau protein amino acid sequences of different animal species.

Species [species, sequence ID]	Amino acid sequence		
Human [*Homo sapiens*, NP_005901.2]	1 MAEPRQEFEVMEDHAGTYGLGDRKD---QGGYTMHQDQEGDTDAGLKESPLQTPTEDGSEEPGSETSDAKSTPTAEDVTAPLVDEGAPGKQAAAQPHTEI	97	
Chimpanzee [*Pan troglodytes*, XP_009430187.1]	1 MAEPRQEFEVMEDHAGTYGLGDRKD---QGGYTMHQDQEGDTDAGLKESPLQTPTEDGSEEPGSETSDAKSTPTAEDVTAPLVDEGAPGKQAAAQPHTEI	97	
Cat [*Felis catus*, XP_003997079.1]	1 MAEPRQDFTVMDDHAGTYGTGERKDLPSQGSYTLMQDHEGDVDQGLKESPLQTPADDGSEEPGSETSDAKSTPTAEDATAPLVDEGAPGEQAAAQPHTEI	100	
Dog [*Canis lupus familiaris*, XP_005624235.1]	1 MAEPRQEFDVMEDHAGTYGLGDRKD---QEGYTMLQDQEGDTDAGLKESPLQTPAEDGSEELGSETSDAKSTPTAEDVTAPLVDERAPGEQAAAQPHMEI	97	
Mouse [*Mus musculus*, NP_001033698.1]	1 MADPRQEFDTMEDHAG-------------DYTLLQDQEGDMDHGLKESPPQPPADDGAEEPGSETSDAKSTPTAEDVTAPLVDERAPDKQAAAQPHTEI	86	
	98 PEGTTAEEAGIGDTPSLEDEAAGHVTQARMVSKSKDGTGSDDKKAKGADGK--TKIATPRGAAPPGQKGQANATRIPAKTPPAPKTPPSSGEPPKSGDRS	195	
	98 PEGTTAEEAGIGDTPSLEDEAAGHVTQARMVSKSKDGTGSDDKKAKGADGK--TKIATPRGAAPPGQKGQANATRIPAKTPPAPKTPPSSGEPPKSGDRS	195	
	101 PEGTTAEEAGIGDTPNLEDQAAGHVTQARMVSKGRDGTGADDKKAKGADGKAGTKIATPRGAAPPGQKGQANATRIPAKTTPSPKTPPGTGDSGKSGDRS	200	
	98 PEGTTAEEAGIGDTPSLEDEAAGHVTQARMVSKSKDGTGSDDKKAKGADGK--TKIATPRGAAPPGQKGQANATRIPAKTPPAPKTPPSSGEPPKSGDRS	195	
	87 PEGITAEEAGIGDTPNQEDQAAGHVTQARVASKD--RTGNDEKKAKGADGKTGAKIATPRGAASPAQKGTSNATRIPAKTTPSPKTPPGSGEPPKSGERS	184	
	MBD1, MBD2		
	196 GYSSPGSPGTPGSRSRTPSLPTPPTREPKKVAVVRTPPKSPSSAKSRLQTAPVPMPDLKNVKSKIGSTENLKHQPGGGKVQIINKKLDLSNVQSKCGSKD	295	
	196 GYSSPGSPGTPGSRSRTPSLPTPPTREPKKVAVVRTPPKSPSSAKSRLQTAPVPMPDLKNVKSKIGSTENLKHQPGGGKVQIINKKLDLSNVQSKCGSKD	295	
	201 GYSSPGSPGTPGSRSRTPSLPTPPTREPKKVAVVRTPPKSPSSAKSRLQTAPVPMPDLKNVRSKIGSTENLKHQPGGGKVQIINKKLDLSNVQSKCGSKD	300	
	196 GYSSPGSPGTPGSRSRTPSLPTPPAREPKKVAVVRTPPKSPSSAKSRLQTAPVPMPDLKNVKSKIGSTENLKHQPGGGKVQIINKKLDLSNVQSKCGSKD	295	
	185 GYSSPGSPGTPGSRSRTPSLPTPPTREPKKVAVVRTPPKSPSASKSRLQTAPVPMPDLKNVRSKIGSTENLKHQPGGGKVQIINKKLDLSNVQSKCGSKD	284	
	MBD2, MBD3, MBD4		
	296 NIKHVPGGGSVQIVYKPVDLSKVTSKCGSLGNIHHKPGGGQVEVKSEKLDFKDRVQSKIGSLDNITHVPGGGNKKIETHKLTFRENAKAKTDHGAEIVYK	395	
	296 NIKHVPGGGSVQIVYKPVDLSKVTSKCGSLGNIHHKPGGGQVEVKSEKLDFKDRVQSKIGSLDNITHVPGGGNKKIETHKLTFRENAKAKTDHGAEIVYK	395	
	301 NIKHVPGGGSVQIVYKPVDLSKVTSKCGSLGNIHHKPGGGQVEVKSEKLDFKDRVQSKIGSLDNITHVPGGGNKKIETHKLTFRENAKAKTDHGAEIVYK	400	
	296 NIKHVPGGGSVQIVYKPVDLSKVTSKCGSLGNIHHKPGGGQVEVKSEKLDFKDRVQSKIGSLDNITHVPGGGNKKIETHKLTFRENAKAKTDHGAEIVYK	395	
	285 NIKHVPGGGSVQIVYKPVDLSKVTSKCGSLGNIHHKPGGGQVEVKSEKLDFKDRVQSKIGSLDNITHVPGGGNKKIETHKLTFRENAKAKTDHGAEIVYK	385	
	396 SPVVSGDTSPRHLSNVSSTGSIDMVDSPQLATLADEVSASLAKQGL	441	homology to human tau 100%
	396 SPVVSGDTSPRHLSNVSSTGSIDMVDSPQLATLADEVSASLAKQGL	441	100%
	401 SPVVSGDTSPRHLSNVSSTGSIDMVDSPQLATLADEVSASLAKQGL	446	93%
	396 SPVVSGDTSPRHLSNVSSTGSIDMVDSPQLATLADEVSASLAKQGL	441	92%
	385 SPVVSGDTSPRHLSNVSSTGSIDMVDSPQLATLADEVSASLAKQGL	430	89%

MBD: microtubule binding domain.
